# Decreasing undesirable absorbed radiation to the intestine after administration of radium-223 dichloride for treatment of bone metastases

**DOI:** 10.1038/s41598-020-68846-x

**Published:** 2020-07-17

**Authors:** Kazuma Ogawa, Takuma Higashi, Kenji Mishiro, Hiroshi Wakabayashi, Kazuhiro Shiba, Akira Odani, Seigo Kinuya

**Affiliations:** 1 Institute for Frontier Science Initiative, Kanazawa University, Kakuma-machi, Kanazawa, 920-1192 Japan; 2 Graduate School of Medical Sciences, Kanazawa University, Kakuma-machi, Kanazawa, 920-1192 Japan; 3 Kanazawa University Hospital, Takara-machi, Kanazawa, 920-8641 Japan; 4 Advanced Science Research Center, Kanazawa University, Takara-machi, Kanazawa, 920-8640 Japan

**Keywords:** Medical research, Drug development

## Abstract

[^223^Ra]RaCl_2_ is the first alpha-particle emitting radiopharmaceutical to be used for castration-resistant prostate cancer patients with bone metastases because of its excellent therapeutic effects. [^223^Ra]RaCl_2_ is excreted via the intestine into feces, and some is absorbed from the intestine into the blood, which may be undesirable in terms of the exposure to radiation. Recently, we showed that a complex of *myo*-inositol-hexakisphosphate (InsP6) with zinc is a useful decorporation agent against radiostrontium. In this study, we hypothesized that Zn-InsP6 could bind to not only strontium but also to radium, and could inhibit the absorption of radium from the intestine. In in vitro binding experiments, Zn-InsP6 showed a high binding affinity for radium. In in vivo biodistribution experiments by intravenous injection of [^223^Ra]RaCl_2_ after treatment of Zn-InsP6, mice treated with Zn-InsP6 showed significantly lower bone accumulation of radioactivity (34.82 ± 1.83%Dose/g) than the mice in the non-treatment control group (40.30 ± 2.78%Dose/g) at 48 h postinjection. These results indicate that Zn-InsP6 bound radium in the intestine and inhibited the absorption of radium into the blood. Therefore, the insoluble Zn-InsP6 complex has high potential to decrease the side effects of [^223^Ra]RaCl_2_.

## Introduction

Most metastatic bone cancers cause severe pain and decrease the patients’ quality of life^1^. Several radiopharmaceuticals have been studied for palliation of metastatic bone pain, and [^89^Sr]SrCl_2_ (Metastron) and [^153^Sm]Sm-EDTMP (Quadramet), which is a complex between ^153^Sm and the calcium salt of ethylenediaminetetramethylene phosphonic acid, have been approved by the Food and Drug Administration (FDA)^2,3^. [^223^Ra]RaCl_2_ (Xofigo) was approved as the first alpha-particle emitting radiopharmaceutical for castration-resistant prostate cancer patients with bone metastases in the US, EU, and many other countries. In a phase III randomized trial (ALSYMPCA), [^223^Ra]RaCl_2_ has shown excellent therapeutic effects, such as prolonged the overall survival and prolonged period to the first symptomatic skeletal event^4^.

After injection of bone-seeking radiopharmaceuticals, such as [^99m^Tc]Tc-bisphosphonate complexes, [^153^Sm]Sm-EDTMP, [^18^F]NaF, and [^89^Sr]SrCl_2_, almost all radioactivity, except that that has accumulated in the bone, is rapidly excreted via the kidney into the urine. On the other hand, after injection of [^223^Ra]RaCl_2_, the main route of excretion is into the feces, and a much higher degree of radioactivity is excreted via the intestine into the feces compared to other bone-seeking radiopharmaceuticals^5^. The mechanism of transport from the blood into the intestine is not well understood^6^. [^223^Ra]Ra^2+^ in the gut should hardly affect the intestinal wall because the range of alpha particles is very short. However, it is known that some [^223^Ra]Ra^2+^ is absorbed from the intestine^7^. The absorption of [^223^Ra]Ra^2+^ may be undesirable from the point of view of the radiation exposure to the intestine wall.

**Figure 1:**
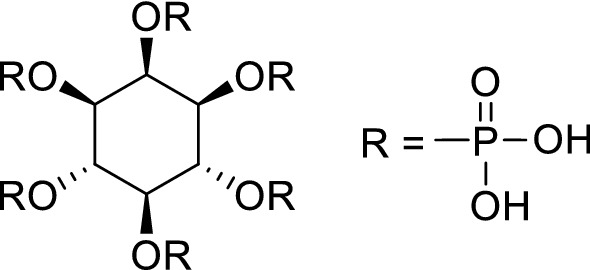
Structure of *myo*-inositol-hexakisphosphate (InsP6).

We have previously conducted studies with the aim to decrease the dose of absorbed radiation in the case of intake incident of radionuclides^8–10^. *Chlorella*, a genus of single-cell green algae that grows in fresh water, is a known health food composed of approximately 1–4% chlorophyll, 55–67% protein, 9–18% dietary fiber, and large amounts of minerals and vitamins^11^. It has been previously reported that *Chlorella* enhances the excretion of heavy metals^12–15^. Thus, we hypothesized that *Chlorella* could also enhance the elimination of radiocesium (Cs^+^) and radiostrontium (Sr^2+^), which are major radionuclides released into the atmosphere and ocean by nuclear power plant accidents, from the body in the case of intake accidents. The study indicated that *Chlorella* could inhibit the absorption of ^90^Sr into the blood and enhance the elimination of ^90^Sr from the body through adsorption in intestine^8^. Moreover*, Myo*-inositol-hexakisphosphate (phytic acid: InsP6, Fig. 1), a natural product found in abundance in plants, especially in whole grains, cereals, legumes, seeds, and nuts^16^, possesses high chelation potential with many kinds of metal cations due to its structure^17–19^. Because Zn-InsP6 is insoluble in water and has enough room to potentially accommodate additional radiocesium or radiostrontium coordinated by chelation, we prepared and evaluated a complex of InsP6 with zinc ions (Zn-InsP6) for use as decorporation agents for radiocesium and radiostrontium. The results demonstrated that Zn-InsP6 adsorbed radiocesium or radiostrontium in vitro. Although Zn-InsP6 did not affect radiocesium in vivo, Zn-InsP6 adsorbed radiostrontium in the gastrointestinal tract, inhibited its absorption from the intestine into the blood, and enhanced its excretion into the feces^9,10^. Accordingly, these studies indicated that *Chlorella* and Zn-InsP6 could work as decorporation agents for radiostrontium.

Strontium and radium are classified as alkaline earth metals and have some similarities in their physical and chemical properties. Thus, we hypothesized that *Chlorella* and Zn-InsP6 could bind not only strontium but also radium, and could inhibit the absorption of radium from intestine into blood. If *Chlorella* and Zn-InsP6 can inhibit the absorption of [^223^Ra]Ra^2+^ from the intestine, it is expected that the side effects of [^223^Ra]RaCl_2_ in the intestine will be decreased by pretreatment with *Chlorella* or Zn-InsP6. Therefore, in this study, we evaluated the effects of *Chlorella* and Zn-InsP6 to [^223^Ra]RaCl_2_ both in vitro and in vivo.

## Results and discussion

### Experiments of in vitro ^223^Ra adsorption at pH 1.2 and pH 6.8 by *Chlorella* and Zn-InsP6

**Figure 2:**
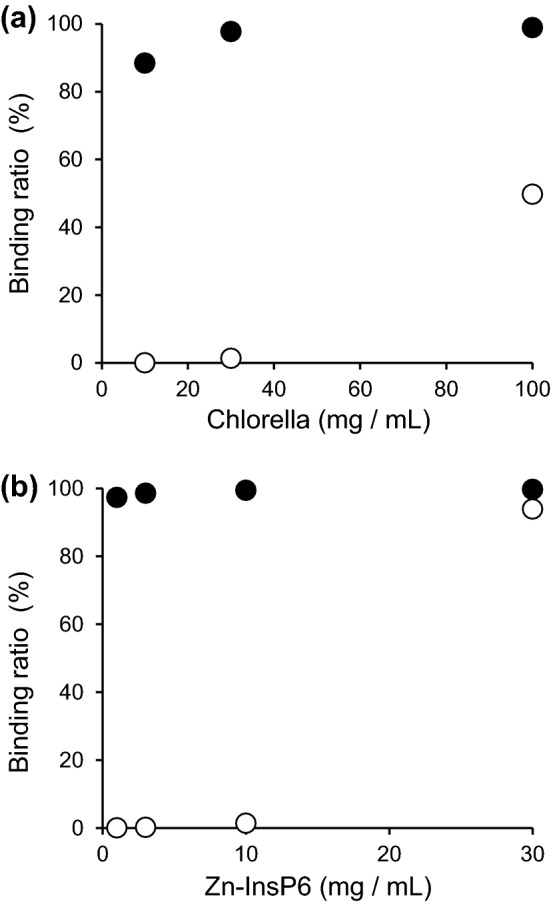
Adsorption of ^223^Ra to (**a**) *Chlorella* or (**b**) Zn-InsP6*.* The binding ratios of ^223^Ra at pH 1.2 (open circles) and pH 6.8 (closed circles) to *Chlorella* or Zn-InsP6 depended on the concentration of *Chlorella* or Zn-InsP6. Data are expressed as the mean ± SD of three samples. The highest SD values in (**a**) and in (**b**) are 0.69 and 1.57, respectively.

The binding ratios of ^223^Ra to *Chlorella* and Zn-InsP6 are shown in Fig. 2. ^223^Ra adsorbed to *Chlorella* in a quantity-dependent manner and the absorption ratios of ^223^Ra (e.g. 97.7% ± 0.0% for 30 mg *Chlorella*/mL) were much higher than those of ^85^Sr (e.g. 35.9% ± 0.9% for 30 mg *Chlorella*/mL) under a neutral pH condition^8^. ^223^Ra highly adsorbed to Zn-InsP6 even at low concentrations under a neutral pH condition. Moreover, ^223^Ra only adsorbed to *Chlorella* and Zn-InsP6 at high concentrations under an acidic pH condition. In this study, the binding affinity for ^223^Ra under an acidic condition that mimics gastric acid is not so important because ^223^Ra does not pass through the stomach after intravenous injection of [^223^Ra]RaCl_2_ as a therapeutic radiopharmaceutical. However, it was necessary to evaluate whether compounds bind radionuclides under acidic conditions in the application for decorporation agents of radionuclides in the case of intake accidents.

### pH dependence of in vitro adsorption of ^223^Ra to *Chlorella* or Zn-InsP6

**Figure 3:**
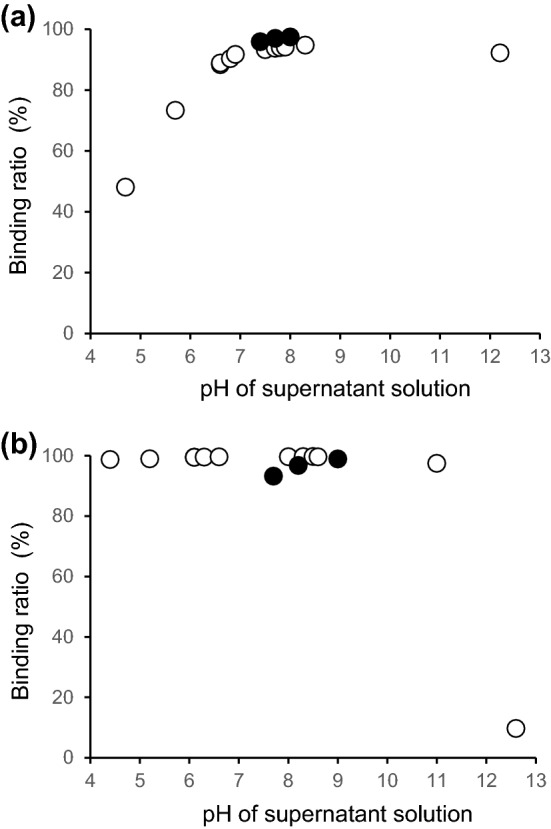
pH dependence of *Chlorella* or Zn-InsP6 adsorption. The binding ratios of ^223^Ra to (**a**) *Chlorella* or (**b**) Zn-InsP6 depended on the pH values in supernatant solution (open circles). The binding ratios of ^223^Ra after exposure to acidic conditions (closed circles). Data are expressed as the mean ± SD of three samples. The highest SD values in (**a**) and in (**b**) are 1.38 and 2.13, respectively.

The open circles in Fig. 3a, b show the pH dependence of ^223^Ra adsorption to *Chlorella* and Zn-InsP6, respectively. The binding ratios of ^223^Ra to *Chlorella* were low at low pH, and the binding ratios increased with increasing pH, and were high (> 90%) at pH between 6.6 and 12.2. Furthermore, the binding ratios of ^223^Ra to Zn-InsP6 were higher at a wider range of pH compared to those to *Chlorella*.

The closed circles in Fig. 3a, b show the binding ratios of ^223^Ra to *Chlorella* and Zn-InsP6 at adjusted neutral pH after the *Chlorella* and Zn-InsP6 samples were exposed to an acidic condition. The results indicated that even after exposure to an acidic solution, *Chlorella* and Zn-InsP6 still highly bound ^223^Ra at neutral pH. Namely, *Chlorella* and Zn-InsP6 after passing through the acidic conditions in the stomach could bind ^223^Ra in the intestine.

### Effects of cations (Na^+^, K^+^, or Ca^2+^) on in vitro adsorption of [^223^Ra]Ra^2+^ by *Chlorella* and Zn-InsP6

**Figure 4:**
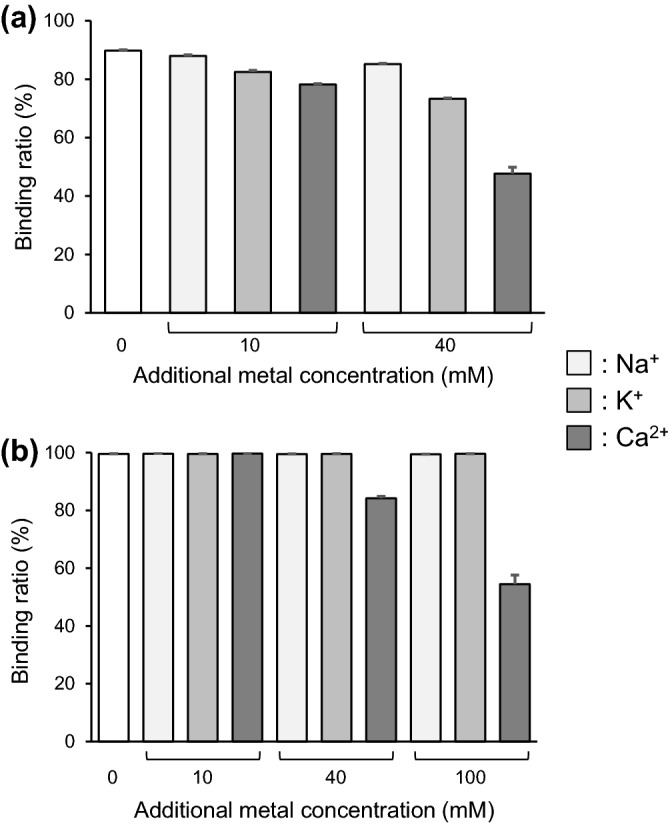
Binding ratios of [^223^Ra]Ra ^2+^ to (**a**) *Chlorella* or (**b**) Zn-InsP6 in the presence of Na^+^, K^+^, or Ca^2+^. Zero additional metal concentration (white columns) means using HEPES buffer containing 10 mM Na^+^. Data are expressed as the mean ± SD of three samples.

Figure 4a, b show how the binding of Ra^2+^ to *Chlorella* and Zn-InsP6 is affected by other cations, Na^+^, K^+^, or Ca^2+^. The presence of Na^+^ and K^+^ slightly decreased the amount of Ra^2+^ bound to *Chlorella*, while there was no decrease in the binding rate of Ra^2+^ in the case of Zn-InsP6. In addition, the presence of Ca^2+^ decreased the binding ratios of Ra^2+^ to both *Chlorella* and Zn-IP6, and this effect was dependent on Ca^2+^ concentration. As Ca^2+^ and Ra^2+^ are divalent cations and classified as alkaline earth metals, it is reasonable that the high concentration of Ca^2+^ inhibits the binding of Ra^2+^.

Based on the abovementioned results of the in vitro experiments, in which Zn-InsP6 showed higher affinity for Ra^2+^ and was expected to be more effective than *Chlorella*, we performed in vivo experiments using only Zn-InsP6 (and not *Chlorella*).

### Biodistribution experiments after administration of [^223^Ra]RaCl_2_ with pretreatment of Zn-InsP6

The hypothesis of this study is that Zn-InsP6 binds [^223^Ra]Ra^2+^ in the intestine and inhibits the absorption of [^223^Ra]Ra^2+^ from the intestine, decreasing the radiation dose to the intestinal wall from ^223^Ra. Thus, although [^223^Ra]RaCl_2_ is used as a radiopharmaceutical for intravenous injection, biodistribution experiments after oral administration of [^223^Ra]RaCl_2_ after pretreatment of Zn-InsP6 were first performed to confirm the binding of Zn-InsP6 with [^223^Ra]Ra^2+^ and the inhibiting absorption of [^223^Ra]Ra^2+^ from the intestine (Table 1). Almost all radioactivity was cleared from almost all tissues, with the exception of the bone (femur), 48 h after oral administration of [^223^Ra]RaCl_2_ in all study groups. Pretreatment with Zn-InsP6 significantly decreased the radioactivity in the bone compared to the non-treatment control group. These results indicate that Zn-InsP6 inhibited the absorption of [^223^Ra]Ra^2+^ from the intestine into the blood, as it is known that [^223^Ra]Ra^2+^ in the blood rapidly and highly accumulates in the bone. The biodistribution of [^223^Ra]RaCl_2_ with pretreatment of InsP6 was also investigated as an additional control group to confirm whether Zinc is essential in the Zn-InsP6 complex as an agent for inhibiting the absorption of [^223^Ra]Ra^2+^. Since InsP6 could bind [^223^Ra]Ra^2+^, InsP6 alone may inhibit the absorption of [^223^Ra]Ra^2+^ from the intestine. The results demonstrated that the bone accumulation of radioactivity in the pretreatment with InsP6 group was similar to that in the non-treatment control group, indicating that InsP6 alone did not inhibit the absorption of [^223^Ra]Ra^2+^. Namely, InsP6 alone, which is water-soluble, did not work as an inhibitor of the absorption of [^223^Ra]Ra^2+^; thus, the water-insolubility of Zn-InsP6 complex is likely to be important for the inhibition of the absorption.

**Table 1:** Biodistribution of radioactivity in mice at 48 h after oral administration of [^223^Ra]RaCl_2_ with pretreatment of 5% glucose (control), InsP6, or Zn-InsP6.

Tissue	Control	InsP6	Zn-InsP6
Blood	0.01 (0.01)	0.01 (0.00)	0.01 (0.00)
Liver	0.01 (0.00)	0.01 (0.01)	0.01 (0.00)
Kidney	0.09 (0.03)	0.14 (0.03)	0.08 (0.05)
Small-intestine^†^	0.10 (0.09)	0.08 (0.02)	0.05 (0.04)
Large-intestine^†^	0.28 (0.22)	0.11 (0.07)	0.09 (0.09)
Spleen	0.30 (0.17)	0.35 (0.16)	0.29 (0.18)
Pancreas	0.03 (0.03)	0.04 (0.03)	0.06 (0.02)
Lung	0.02 (0.01)^#^	0.06 (0.02)*	0.02 (0.01)
Heart	0.02 (0.01)	0.04 (0.01)	0.04 (0.03)
Stomach^†^	0.20 (0.31)	0.06 (0.02)	0.07 (0.11)
Bone (Femur)	12.06 (4.60)*	12.20 (4.32)*	4.38 (3.76)
Muscle	0.03 (0.03)	0.04 (0.02)	0.07 (0.07)
Brain	0.03 (0.01)	0.04 (0.03)	0.32 (0.67)

Data are expressed as % injected dose per gram tissue. Each value represents the mean (SD) for from four to seven animals.

^†^Data are expressed as % injected dose.

Significance of multiple comparison was determined using a one-way ANOVA followed by Tukey’s post hoc test (*p < 0.05 vs. Zn-InsP6, ^#^*p* < 0.05 vs. InsP6).

Next, the effects of pretreatment with Zn-InsP6 on the biodistribution at 1, 24, and 48 h after intravenous injection of [^223^Ra]RaCl_2_ were evaluated and the results are shown in Table 2. After intravenous injection of [^223^Ra]RaCl_2_, a large portion of the radioactivity immediately accumulated in the bone, while the remainder was delivered to the kidney and intestine. At 1 h postinjection, the radioactivity in all tissues was almost the same between the Zn-InsP6 treated group and the non-treatment control group. Thus, although the decrease in the bone accumulation following pretreatment with Zn-InsP6 was not so substantial, it was significant at 48 h postinjection. Moreover, the excreted radioactivity into the feces was significantly increased. These results are in line with the abovementioned biodistribution after oral administration of [^223^Ra]RaCl_2_ and indicate that Zn-InsP6 bound [^223^Ra]Ra^2+^ in the intestine and inhibited the absorption of [^223^Ra]Ra^2+^ from the intestine.

**Table 2:** Biodistribution of radioactivity in mice at 1, 24, and 48 h after intravenous administration of [^223^Ra]RaCl_2_ with pretreatment of 5% glucose (control) or Zn-InsP6.

	1 h	24 h	48 h
**Control tissue**			
Blood	0.35 (0.03)	0.04 (0.02)	0.03 (0.04)
Liver	0.43 (0.04)	0.05 (0.01)	0.01 (0.01)
Kidney	10.66 (0.55)	0.92 (0.15)	0.25 (0.01)
Small-intestine^†^	7.03 (0.63)	0.91 (0.49)	0.10 (0.01)
Large-intestine^†^	1.41 (0.29)	4.38 (2.32)	0.24 (0.17)
Spleen	3.48 (0.71)	0.93 (0.28)	1.04 (0.76)
Pancreas	0.37 (0.08)	0.06 (0.02)	0.08 (0.14)
Lung	1.12 (0.09)	0.11 (0.08)	0.03 (0.02)
Heart	0.37 (0.03)	0.04 (0.01)	0.01 (0.02)
Stomach^†^	1.98 (0.52)	0.26 (0.12)	0.03 (0.01)
Bone (Femur)	31.03 (4.09)	39.73 (4.10)	40.30 (2.78)
Muscle	0.31 (0.02)	0.10 (0.14)	0.06 (0.07)
Brain	0.08 (0.02)	0.08 (0.03)	0.10 (0.04)
Feces^†^			8.08 (2.00)
Urine^†^			12.22 (4.92)
**Zn-InsP6 tissue**			
Blood	0.39 (0.09)	0.03 (0.00)	0.01 (0.00)
Liver	0.44 (0.12)	0.04 (0.02)	0.01 (0.00)
Kidney	12.67 (2.57)	0.87 (0.18)	0.23 (0.06)
Small-intestine^†^	7.64 (0.89)	1.06 (1.03)	0.09 (0.01)
Large-intestine^†^	1.59 (0.35)	5.46 (2.30)	0.15 (0.05)
Spleen	4.36 (1.61)	1.22 (0.89)	0.69 (0.22)
Pancreas	0.42 (0.05)	0.07 (0.04)	0.03 (0.03)
Lung	1.16 (0.15)	0.13 (0.09)	0.04 (0.03)
Heart	0.38 (0.08)	0.07 (0.02)	0.02 (0.01)
Stomach^†^	1.55 (0.33)	0.92 (0.98)	0.04 (0.01)
Bone (Femur)	31.51 (4.86)	35.75 (3.42)	34.82 (1.83)*
Muscle	0.45 (0.20)	0.13 (0.06)	0.04 (0.04)
Brain	0.07 (0.02)	0.11 (0.06)	0.09 (0.04)
Feces^†^			14.28 (2.56)*
Urine^†^			11.26 (2.43)

Data are expressed as % injected dose per gram tissue. Each value represents the mean (SD) for four or five animals.

^†^Data are expressed as % injected dose.

Significance was determined using an unpaired Student’s *t*-test (*p < 0.05 vs. control).

In a previous Phase I study for dosimetry of [^223^Ra]RaCl_2_, the absorbed doses of the small intestinal wall, the upper large intestinal wall, and the lower large intestinal wall were set to 0, derived from alpha-particles^6^. It was assumed that all the radioactivity in the intestine was in the intestinal contents, and that the contribution of alpha-particles from the intestinal contents to the walls was negligible, as stated by International Commission on Radiological Protection (ICRP) publication 100^20^. The absorbed radiation dose to the intestine during absorbing ^223^Ra though the intestine was not considered in the Phase I study. Actually, in a proposed compartmental model for ^223^Ra by Taprogge et al., ^223^Ra was excreted into faces without the reabsorption from the small intestine^21^. However, some ^223^Ra in the intestine must be absorbed into the blood, and the alpha-particles emitted from ^223^Ra on absorption should, in theory, harm the intestinal cells. Indeed, diarrhea has been reported in some patients of clinical studies of [^223^Ra]RaCl_2_^6,22–24^. Classically, the contamination by people painting watch dials with radium from the 1910′s to the 1920′s suggests the absorption of radium because the distribution of radium to the bone of the dial painters had been found^25^. Epidemiological studies of radium dial painters also found an association between exposure to high-dose radium and osteosarcoma^26^. ICRP Task Group on Alkaline Earth Metabolism in Adult Man indicated that the fraction of radium absorbed from food or drinking-water is 0.15–0.21^27^. The absorbed fraction of radium was set to 0.2 in some reports^28^. Moreover, in the above-mentioned Phase I study, Chittenden et al. reported that cumulative urine excretion of ^223^Ra was 2% ± 2% of the injected activity^6^. In another Phase I study in Japan, Yoshida et al. reported that the cumulative urine excretion reached 2% up to 48 h postinjection^29^. These results showed that the urine excretion of ^223^Ra in humans negligible. Meanwhile, some radioactivity in the kidney at 1 h postinjection and in the urine at 48 h postinjection were observed in this study using mice. The difference should be caused by species differences. Thus, the strategy of this study with Zn-InsP6 complex could be more useful to humans because the fecal excretion rate of ^223^Ra in humans must be higher than that in mice. Therefore, the inhibition of the absorption by binding of the insoluble Zn-InsP6 complex with [^223^Ra]Ra^2+^ is considered to be useful in decreasing side effects.

We supposed that the toxicity of Zn-InsP6 is low since the Zn-InsP6 complex is not absorbed due to its insolubility in water, similarly to barium sulfate, which is used as a contrast agent for X-ray diagnosis. If a small part of Zn-InsP6 is decomposed and absorbed into the body, InsP6 and Zn are known to be low toxicity compounds. InsP6 also exists in mammals, and works as a coenzyme^30^. InsP6 is orally taken as an over-the-counter supplement, and brings several health benefits without toxicity, and its anticancer efficacy has also been reported^31^. Zinc is an essential mineral and is also used as an over-the-counter supplement that is recommended for individuals at a high risk of zinc deficiency, which is associated with a number of diseases^32^. Namely, because Zn-InsP6 is a combination of a well-known element and a compound that are both commonly taken as supplements, it is likely that Zn-InsP6 will be suitable for use in clinical research.

## Conclusions

Zn-InsP6 showed an excellent binding affinity for ^223^Ra, and pretreatment with Zn-InsP6 was shown to decrease radioactivity in the bone after administration of [^223^Ra]RaCl_2_. These results indicate that the insoluble Zn-InsP6 complex bound [^223^Ra]Ra^2+^ in the intestine and inhibited the absorption of [^223^Ra]Ra^2+^ from the intestine into the blood. Thus, Zn-InsP6 has good potential for decreasing side effects of [^223^Ra]RaCl_2_.

## Methods

### Materials

[^223^Ra]RaCl_2_ was obtained from Bayer Yakuhin, Ltd (Osaka, Japan). Chlorella powder was supplied by Daesang Corp. (Seoul, Korea). InsP6 was purchased from Sigma-Aldrich (St. Louis, MO, USA) as phytic acid sodium salt hydrate, InsP6·6Na^+^·6H_2_O. Other reagents were of reagent grade and were used as received.

### In vitro adsorption of ^223^Ra to *Chlorella* and Zn-InsP6

Zn-InsP6 was prepared by the method previously reported with slight modification^9^. Zn-InsP6 (Zn : InsP6 = 2 : 1) was used for both in vitro and in vivo experiments. This in vitro adsorption experiment was performed according to our previous study^8^. Namely, *Chlorella* (10, 30, or 100 mg) or Zn-InsP6 (1, 3, 10, or 30 mg) was suspended and ^223^Ra (925 Bq) was added in 1 mL of the first test solution (artificial gastric juice, pH 1.2) or the second test solution (artificial intestinal juice, pH 6.8) defined in the Japanese Pharmacopoeia. After shaking the suspension at 1,000 rpm at 37ºC for 1 h using a shaking incubator (SI-300C; AS ONE Corp., Osaka, Japan), the samples were centrifuged at 10,000* g* at room temperature for 10 min. The radioactivity of the supernatant was measured using an auto-well gamma counter (ARC-7010B; Hitachi Ltd., Tokyo, Japan) and the counts were corrected for background radiation. A window from 50 to 300 keV was used for the counting. The measurement time of each sample was set to 1 min. Control experiments were performed using the same procedure but without *Chlorella* or Zn-InsP6. The binding ratios were determined as follows:

Binding ratio to *Chlorella* or Zn-InsP6 (%) = [1 − (radioactivity of the supernatant of each sample) / (radioactivity of the supernatant of the respective control)] × 100.

### pH dependence of the in vitro adsorption of ^223^Ra to *Chlorella* or Zn-InsP6

This experiment was also performed according to our previous study with slight modification^8^. Namely, *Chlorella* (30 mg) or Zn-InsP6 (10 mg) was suspended and ^223^Ra (925 Bq) was added in 1 mL of 0.01 M HEPES buffer solution (pH 2–13) and shaking, centrifugation, and radioactivity measurements were performed as described above. The pH of each suspension was measured after shaking.

Reversibility of the adsorption potential between *Chlorella* or Zn-InsP6 and ^223^Ra with pH variation was evaluated. *Chlorella* (30 mg) or Zn-InsP6 (10 mg) was suspended in 1 mL of the first test solution (pH 1.2) defined in the Japanese Pharmacopoeia and then shaken at 1,000 rpm at 37ºC for 1 h. After centrifugation at 10,000* g* at room temperature for 10 min, 800 μL of the supernatant was removed. Following this, 23, 25, or 27 µL of 1 M NaOH solution and 777, 775, or 773 µL of 0.01 M HEPES buffer solution (pH 8) were added to the *Chlorella* suspension. Meanwhile, 9, 11, or 13 µL of 1 M NaOH solution and 791, 789, or 787 µL of 0.01 M HEPES buffer solution (pH 8) were added to the InsP6 suspension. Thereafter, ^223^Ra (925 Bq) was added to the *Chlorella* or InsP6 suspension, and the suspension was shaken at 1,000 rpm and 37ºC for 1 h. After centrifugation at 10,000* g* at room temperature for 10 min, the radioactivity and pH of the supernatant were measured as described above.

### Effects of cations (Na^+^, K^+^, or Ca^2+^) on the in vitro adsorption of ^223^Ra to *Chlorella* or Zn-InsP6

This experiment was performed according to our previous study with slight modification ^10^. ^223^Ra solutions (925 Bq/mL) in 20 mM HEPES buffer (pH 7.4) containing 10 mM Na^+^, 20 mM Na^+^, 50 mM Na^+^, 110 mM Na^+^ (in the case of Zn-InsP6), 10 mM Na^+^  + 10 mM K^+^, 10 mM Na^+^  + 40 mM K^+^, 10 mM Na^+^  + 100 mM K^+^ (in the case of Zn-InsP6), 10 mM Na^+^  + 10 mM Ca^2+^, 10 mM Na^+^  + 40 mM Ca^2+^, and 10 mM Na^+^  + 100 mM Ca^2+^ (in the case of Zn-InsP6) were prepared by dissolution of NaCl, KCl, or CaCl_2_. *Chlorella* (10 mg) or Zn-InsP6 (10 mg) was suspended in 1 mL of each ^223^Ra solution. Additionally, after shaking the suspensions at 1,000 rpm at 37ºC for 1 h, the binding ratio of each sample to *Chlorella* or Zn-InsP6 was determined using the methods described above.

### Biodistribution experiments after oral administration of ^223^Ra with pretreatment of Zn-IP6

Animal experiments were conducted in strict accordance with the Guidelines for the Care and Use of Laboratory Animals of Kanazawa University. The animal experimental protocols were approved by the Committee on Animal Experimentation of Kanazawa University. The animals were housed with free access to food and water at 23ºC with a 12-h alternating light/dark schedule unless otherwise specified.

6-week-old male ddY mice (Japan SLC Inc., Hamamatsu, Japan) were used for all animal experiments. In the Zn-InsP6 administration group, Zn-InsP6 suspension (30 mg/0.5 mL) in 5% glucose aqueous solution was orally administrated into mice. In the InsP6 administration group, InsP6 solution (20 mg/0.5 mL) in 5% glucose was orally administrated into mice. In the control group, 0.5 mL of 5% glucose was orally administrated into mice. Then, just after the administration of Zn-InsP6, InsP6, or 5% glucose, a saline solution of [^223^Ra]RaCl_2_ (9.25 kBq/100 µL) was orally administrated. Mice were sacrificed at 48 h post-administration of [^223^Ra]RaCl_2_. The tissues of interest were removed and weighed, and radioactivity counts were determined. Fasting was carried out from 12 h pre-administration to 24 h post-administration to exclude the effects of diet.

### Biodistribution experiments after intravenous administration of ^223^Ra after pretreatment with Zn-IP6

In the Zn-InsP6 administration group, Zn-InsP6 suspension (30 mg/0.5 mL) in 5% glucose aqueous solution was orally administrated into 6-week-old male ddY mice. In the control group, 0.5 mL of 5% glucose was orally administrated. Then, 1 h after the administration of Zn-InsP6 suspension or 5% glucose, a saline solution of [^223^Ra]RaCl_2_ (9.25 kBq/100 µL) was injected via the tail vein. To determine the amount and routes of the radioactivity excreted from the body, mice were housed in metabolic cages (Metabolica, Sugiyama gene, Tokyo, Japan) for 48 h after administration. Mice were sacrificed at 1, 24, and 48 h post-administration of [^223^Ra]RaCl_2_. The tissues of interest were removed and weighed, and radioactivity counts were determined with an auto well gamma counter as described above. Fasting was carried out from 12 h pre-administration to 24 h post-administration to exclude the effects of diet.

### Statistical analysis

Significance in biodistribution experiments was determined by a one-way analysis of variance followed by Tukey’s post hoc test or unpaired Student's *t*-test using Prism 8 (GraphPad Software Inc., San Diego, CA, USA). Results were considered statistically significant at *p* < 0.05.
